# Alpha-synuclein is involved in manganese-induced spatial memory and synaptic plasticity impairments via TrkB/Akt/Fyn-mediated phosphorylation of NMDA receptors

**DOI:** 10.1038/s41419-020-03051-2

**Published:** 2020-10-08

**Authors:** Zhuo Ma, Kuan Liu, Xin-Ru Li, Can Wang, Chang Liu, Dong-Ying Yan, Yu Deng, Wei Liu, Bin Xu

**Affiliations:** Department of Environmental Health, School of Public Health, China Medical University, No. 77 Puhe Road, Shenyang North New Area, 110122 Shenyang, Liaoning Province People’s Republic of China

**Keywords:** Neurodegeneration, Hippocampus

## Abstract

Manganese (Mn) overexposure produces long-term cognitive deficits and reduces brain-derived neurotrophic factor (BDNF) in the hippocampus. However, it remains elusive whether Mn-dependent enhanced alpha-synuclein (α-Syn) expression, suggesting a multifaceted mode of neuronal toxicities, accounts for interference with BDNF/TrkB signaling. In this study, we used C57BL/6J WT and α-Syn knockout (KO) mice to establish a model of manganism and found that Mn-induced impairments in spatial memory and synaptic plasticity were related to the α-Syn protein. In addition, consistent with the long-term potentiation (LTP) impairments that were observed, α-Syn KO relieved Mn-induced degradation of PSD95, phosphorylated CaMKIIα, and downregulated SynGAP protein levels. We transfected HT22 cells with lentivirus (LV)-α-Syn shRNA, followed by BDNF and Mn stimulation. In vitro experiments indicated that α-Syn selectively interacted with TrkB receptors and inhibited BDNF/TrkB signaling, leading to phosphorylation and downregulation of GluN2B. The binding of α-Syn to TrkB and Fyn-mediated phosphorylation of GluN2B were negatively regulated by BDNF. Together, these findings indicate that Mn-dependent enhanced α-Syn expression contributes to further exacerbate BDNF protein-level reduction and to inhibit TrkB/Akt/Fyn signaling, thereby disturbing Fyn-mediated phosphorylation of the NMDA receptor GluN2B subunit at tyrosine. In KO α-Syn mice treated with Mn, spatial memory and LTP impairments were less pronounced than in WT mice. However, the same robust neuronal death was observed as a result of Mn-induced neurotoxicity.

## Introduction

Manganese (Mn) is an essential nutrient because it is required for cellular function in many metabolic pathways. However, the central nervous system (CNS) is highly vulnerable to Mn toxicity, which leads to many sensory disturbances and neurobehavioral deficiency. Mn toxicity can also manifest as a disease called manganism, which shares symptoms that are similar to those of Parkinson’s disease (PD)^1^. Manganism is characterized by rigidity, bradykinesia, emotional instability, and intellectual and memory impairment at an early stage. Environmental and occupational Mn exposure occurs mainly by welding, mining, smelters, lacquers, dry cell batteries, and Mn-rich agrochemicals^2^. Bailey^3^ reported that Mn overexposure during development produces long-term cognitive deficits and reduces brain-derived neurotrophic factor (BDNF) in the hippocampus. Although the neurotoxic mechanisms of Mn such as oxidative stress, energy failure, and the disturbance of neurotransmitter metabolism are well-elucidated^4^, the mechanism underlying these spatial learning and memory deficits is unclear.

BDNF, acting through the receptor tropomyosin kinase receptor B (TrkB), is considered a key molecule in the neurobiological mechanisms involved in learning and memory^5^. BDNF binding to TrkB triggers its dimerization through conformational changes and autophosphorylation of tyrosine residues in its intracellular domain (ICD), activating mitogen-activated protein kinase (MAPK), phosphatidylinositol 3-kinase (PI3K), and phospholipase C-g1 (PLC-γ1) signaling pathways, and mediating neural differentiation, survival, and neurogenesis^6^. However, Kang^7^ found that α-synuclein (α-Syn), when overexpressed, selectively interacts with TrkB receptors and inhibits BDNF/TrkB signaling, leading to dopaminergic neuronal death. α-Syn localizes to presynaptic terminals nearby synaptic vesicles and is predominantly expressed in the neurons of the CNS. Our previous research found that Mn-dependent enhanced α-Syn expression leads to neuronal injury^8^. In addition, Mn exposure promotes α-Syn secretion in exosomal vesicles. The overexpression of α-Syn can disrupt synaptic function, resulting in cognitive disturbance^9^. Therefore, we speculate that the overexpression of α-Syn and its subsequent interference with BDNF/TrkB signaling may play an important role in linking the complicated neurobehavioral deficiency observed following Mn exposure, although the molecular mechanism of this process remains elusive.

In this study, we provided evidence that Mn-induced spatial learning and memory deficits are related to α-Syn disturbing the BDNF/TrkB neurotrophic signaling pathways in vivo and in vitro. Additionally, we used shRNA to knockdown α-Syn and found that BDNF/TrkB signaling pathways exhibited a certain recovery. Hence, this innovative finding provides insight into a pathological role(s) of α-Syn in mediating BDNF/TrkB signal transduction and may represent a relatively unknown mechanism by which Mn-dependent enhanced α-Syn expression is responsible for learning and memory impairments in mice.

## Materials and methods

### Chemicals

Manganese (II) chloride tetrahydrate, BDNF, and DuoLink proximity ligation assay (PLA) reagent were purchased from Sigma Chemical Co. (St. Louis, MO, USA). The pGCSIL-GFP-α-Syn small hairpin RNA (shRNA)^10^ was purchased from Shanghai GeneChem Co. Ltd. (Shanghai, China). A Cell Counting Kit-8 (CCK-8) assay kit and lactate dehydrogenase (LDH) kit were purchased from Beyotime Biotech Co. Ltd. (China). α-Syn primary antibody was obtained from Thermo Fisher Scientific. PSD95, SynGAP, β-actin, phospho-CaMKII (Thr286), CaMKII-α, NMDA receptor 2A (GluN2A), NMDA receptor 2B (GluN2B), Fyn, phospho-NMDA receptor 2A (GluN2A) (Tyr1246), and phospho-NMDA receptor 2B (GluN2B) (Tyr1472) primary antibodies were purchased from Cell Signaling Technology. TrkB, AKT, and AKT-phospho-S473 primary antibodies were purchased from Proteintech Biotech Co. Ltd. (China). BDNF and phospho-Fyn (Y530) were purchased from Abcam Ltd. (Hong Kong). Secondary antibodies including horseradish peroxidase (HRP)-conjugated anti-rabbit and HRP-conjugated anti-mouse were purchased from Abcam Ltd. (Hong Kong).

### Animals and treatments

Homozygous α-Syn gene knockout (KO) male mice were crossed with wild-type (WT) female mice (C57BL/6J) (Liaoning Changsheng Biotechnology Co., Ltd. SCXK 2015–0003; Benxi City, China) maintained in a stable breeding colony^8^.

Ten-week-old homozygous WT and α-Syn KO mice (25 ± 5 g) from an identical offspring generation of F7 heterozygotes were used in the in vivo experiments. The WT and α-Syn KO mice were consolidated and then randomly divided into four groups (*n* = 10 per group) (female: male = 1:1): WT control, KO control, WT Mn treatment (100 μmol/kg MnCl_2_), and KO Mn treatment (100 μmol/kg MnCl_2_). The control group mice were intraperitoneally (i.p.) injected with normal saline. MnCl_2_-treated mice were i.p. injected with 100 μmol/kg MnCl_2_·4H_2_O in normal saline five times per week consecutively for 6 weeks. Animals were housed in conventional enclosures at 24 ± 1 °C under a 12 h light/dark cycle. Six mice per group were chosen as the minimal number of animals needed for carrying out a comparative study. Blinding was performed in the experimental procedures and the data analysis.

### Morris water maze (MWM) test

The MWM test was performed to estimate the capacity of spatial learning and memory, as previously described^11^. The test was composed of a circular tank (120 cm in diameter, 50 cm in height) filled with 24 °C water colored with white powder, and an escape platform placed 1.5 cm below the water surface. Some geometric images were fixed around the tank to assist the swimming mice with their orientation, and a video camera monitored the trajectory and transmitted the data to a computer. The mice were placed into the pool from any quadrant, and they were able to swim freely until they reached the platform. This was repeated four times per day, and after 6 days of training, latency, mean speed, and mean distance were analyzed in a probe test phase, and speed, crossing times, and time in the target quadrant were acquired after 48 h.

### Electrophysiology

The mouse brains were carefully placed in the chilled artificial cerebral spinal fluid (aCSF) buffer, including 124 mM NaCl, 3 mM KCl, 26 mM NaHCO_3_, 2 mM CaCl_2_, 1 mM MgSO_4_, 1.25 mM KH_2_PO_4_, and 10 mM D-glucose with 95% O_2_ and 5% CO_2_ gas. Then, the hippocampus was cut into 300-nm thick slices and incubated in aCSF buffer for 2 h. These brain slices were secured with a metal probe in an 8 × 8 array microelectrode chamber, and a MED64 Mobius (Alpha MED Scientific, Japan) microelectrode array system (MEA) was used to record the long-term potentiation (LTP). The test-stimulation intensity was placed in the CA1–CA3 region and set to elicit 30% (or 50%) of the maximum field excitatory postsynaptic potential (fEPSP) response according to the I/O (input/output) curve. After 20 min, the baseline was recorded, and LTP was induced by a 100 Hz/s high-frequency stimulus that was repeated three times, and the fEPSP slope change was recorded for another 60 min^12^.

### Nissl staining

After internal fixation with 4% formaldehyde overnight, the mouse brains were quickly removed and fixed with paraformaldehyde for 3 days. Then, the samples were transferred into 70%, 80%, 90%, 95%, and 100% ethyl alcohol (12 h for each) and dimethylbenzene for 5 min for dehydration. The treated brains were embedded into the paraffin and sliced into 8-µm-thick hippocampus sections. After dewaxing and hydration, these slices were stained with toluidine blue stain (DK0023, Beijing Leagene Biotechnology Co., Ltd.) for 30 min in a dark and humid box at 60 °C. Finally, slices were immersed in 95% ethanol for 1 min and dimethylbenzene for 15 min and sealed with neutral balsam. The slices were observed with a ScanScope scanner system (view magnification: ×400; Leica, USA) and analyzed by ImageScope 12.0^13^.

### Transmission electron microscopy

The neural synapse ultrastructure was observed by transmission electron microscopy according to a protocol from a previous study^14^. Briefly, the brain was fixed in 2.5% glutaraldehyde for 2 h and then postfixed in 1% osmium tetroxide for another 2 h. After dehydration in ethyl alcohol gradients, the sample was embedded, sectioned, and stained with uranyl acetate and lead citrate. Finally, a transmission electron microscope (H-7650, Hitachi) was used for observation at ×40,000 magnification. The thickness of postsynaptic density was analyzed using image analysis software (ImageJ 1.42q, USA).

### Measurement of Mn concentration

The hippocampus was dissected from the mouse brain, weighed, and digested in 500 μl 70% HNO_3_ for the metal-track assay. After partial evaporation, the samples were cooled to room temperature, 500 μl of H_2_O_2_ (36.5–38.0% for trace metal analysis) was added, and the solution was completely evaporated. Then, the precipitate was dissolved in 5 mL of deionized water and analyzed on a Hitachi 180−80 atomic absorption spectrophotometer (Hitachi Ltd., Tokyo, Japan)^14^.

### Cell culture and treatments

Mouse hippocampal neuronal cell line HT22 was purchased from the Shanghai iCell Biotechnology Company. HT22 cells were cultured in Dulbecco’s modified Eagle’s medium (DMEM, Gibco, Cat. no. 10566016) with 10% fetal bovine serum maintained in a humidified atmosphere with 95% air and 5% CO_2_ at 37 °C. Then, cells were differentiated in DMEM medium containing 1× N2 supplement, 50 ng/ml nerve growth factor-β (NGF-β, Gibco, Cat. no. 17502001), 100 μM phorbol 12,13-dibutyrate (Sigma-Aldrich, Cat. no. P1269), and 100 μM dibutyryl cAMP (Millipore, Cat. no. 28745) for 24 h before use^15^. For knockdown of α-Syn, shRNA was designed (Gene Bank Accession No: AF179273)^16^, and lentiviral vector construction and transfection were performed as previously described^17^. The in vitro experiment was divided into three parts, of which the first part was set up with six groups as follows: three control groups (DMEM control, lentivirus (LV)-Syn shRNA group, and LV-scrambled shRNA group) and three 100 µM Mn-treated groups (Mn group, LV-Syn shRNA +Mn group, and LV-scrambled shRNA +Mn group). For the second part, pretreatment with BDNF was performed. Five groups were divided as follows: control group; Mn (100 µM) group; BDNF (80 ng/ml) control group; BDNF (40 ng/ml) +Mn group; and BDNF (80 ng/ml) +Mn group. For the last part, we combined the first part with the second part. HT22 cells and HT22 cells transfected with LV-Syn shRNA were divided into the control group, Mn (100 µM) group, BDNF (80 ng/ml) group, and BDNF (80 ng/ml) +Mn group.

### Cell viability and LDH assay

The cellular viability was evaluated by the CCK-8 assay (CCK-8 Kit, Beyotime, No: C0037, China), and the level of LDH release induced by MnCl_2_ was evaluated by assay with an LDH Kit (Beyotime, No: C0016, China). HT22 cells were seeded onto 96-well plates and treated with 100 µM Mn or pretreated with LV-Syn shRNA, LV-scrambled shRNA, or BDNF. After 24 h of culture, the cells were assayed with the CCK-8 and LDH kits according to the manufacturer’s protocol using a microplate reader (Biotech Synergy H1, USA).

### Western blot analysis

The expression of the protein was evaluated by immunoblotting according to a previously published method^18^. Briefly, total proteins were extracted from the hippocampus and HT22 cells according to standard procedures. Quantified protein (20 µg) was separated via 8%, 10%, or 15% sodium dodecyl sulfate-polyacrylamide gel electrophoresis (SDS-PAGE). Then, the samples were transferred to PVDF membranes, blocked in 5% bovine serum albumin (BSA) for 2 h, and incubated with the appropriate primary antibodies overnight at 4 °C. These important primary antibodies are listed as follows: PSD95 (1:1000, Cell Signaling Technology, Cat. no. #2507), SynGAP (1:1000, Cell Signaling Technology, Cat. no. #3200), TrkB (1:500, Proteintech, Cat. no. 13129), alpha-Synuclein (1:500, Invitrogen, Cat. no. #MA5-12272), β-actin (1:1000, Cell Signaling Technology, Cat. no. #8457), BDNF (1:1000, Abcam, ab182199), CaMKII-α (1:1000, Cell Signaling Technology, Cat. no. #3357), Phospho-CaMKII (Thr286) (1:1000, Cell Signaling Technology, Cat. no. #12716), NMDA Receptor 2A (GluN2A) (1:1000, Cell Signaling Technology, Cat. no. #4205), NMDA Receptor 2B (GluN2B) (1:1000, Cell Signaling Technology, Cat. no. #4207), AKT (1:500, Proteintech, Cat. no. 10176), AKT-phospho-S473 (1:3000, Proteintech, Cat. no. 66444), Fyn (1:1000, Cell Signaling Technology, Cat. no. #4023), Anti-Fyn (phospho Y530) (1:500, Abcam, ab182661), Phospho-NMDA Receptor 2A (GluN2A) (Tyr1246) (1:1000, Cell Signaling Technology, Cat. no. #4206), and Phospho-NMDA Receptor 2B (GluN2B) (Tyr1472) (1:1000, Cell Signaling Technology, Cat. no. #4208). Subsequently, the membranes were incubated with matched secondary antibodies for 2 h. The results were detected using enhanced chemiluminescence (ECL) and analyzed using image analysis software (ImageJ 1.42q, USA).

### Alpha-synuclein western blots

Briefly, after extracted and normalized the total protein from the hippocampus, the samples without boiling were separated by 4–20% non-denaturing polyacrylamide-gradient gel electrophoresis (Thermo Fisher Scientific, USA). Then, the samples were transferred to PVDF membranes and incubated with the alpha-Synuclein antibody (1:500, Invitrogen, Cat. no. #MA5-12272). After 24 h, the membranes were incubated with a matched secondary antibody for 2 h. The results were detected using ECL and analyzed using image analysis software (ImageJ 1.42q, USA).

### Co-immunoprecipitation (Co-IP) of alpha-synuclein with TrkB

HT22 cells were harvested in RIPA buffer with 1 mM phenylmethylsulfonyl fluoride (PMSF) and centrifuged at 12,000×*g* for 15 min at 4 °C. Then, the protein was normalized and mixed with 4 μl of anti-alpha-synuclein antibody (Invitrogen, Cat. no. MA5-12272) or rabbit IgG antibody at 4 °C. The following day, 25 μl Protein A/G magnetic beads were reacted with the sample/antibody complex for 2 h at 4 °C. The supernatant was separated by 8% or 12% SDS-PAGE, and then analyzed by western blot assay^18^. The primary antibody TrkB was purchased from Proteintech Biotech Co. Ltd (China, Cat. no. 13129).

### Proximity ligation assay

Pretreated HT22 cells were assayed using DuoLink PLA according to the manufacturer’s protocol^19^. Negative control sections were prepared using mouse IgG for α-Syn and rabbit immunoglobulin fraction for TrkB. Then, the signal strength of the red points in each cell was calculated and analyzed using a confocal microscope (excitation: 594 nm, emission: 624 nm, magnification: ×60). NIS-Elements Viewer 4.50 software (Nikon, Japan) was used to measure the signaling level.

### Statistical analysis

Statistical analyses were performed with GraphPad Prism 8.0.1 (GraphPad Software, CA, USA). Two-way ANOVA and the SNK-q were used to assess the effect of the Mn factor and genotype factor in vivo. For the in vitro experiments, one-way ANOVA and the SNK-q test were used to analyze the significant variances: *P* values of less than 0.01 or 0.05 were set as statistically significant. All experiments were repeated at least three replicated times. “n” refers to samples obtained from different mice in vivo, and parallel samples in vitro. Bars represent mean ± SD.

## Results

### Mn impairs spatial memory and synaptic plasticity in WT and α-Syn KO mice

**Fig. 1:**
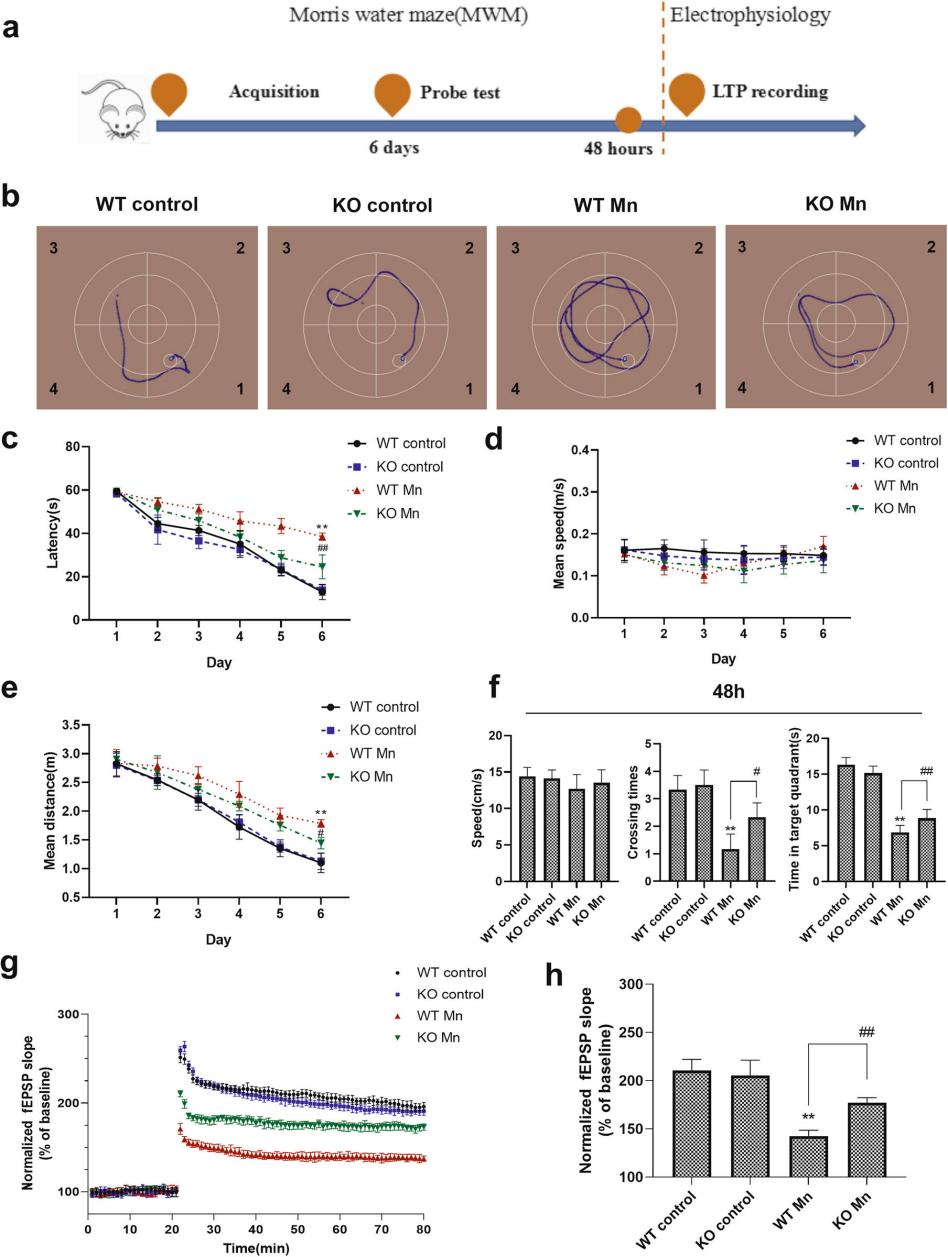
Mn impairs the spatial memory in WT and α-Syn KO mice. **a** The experimental procedure for Morris water maze (MWM) and electrophysiology tests. **b** A representative searching trace on the 6th day of the training phase. **c**–**e** The escape latency, mean speed, and mean distance to reach the hidden platform. **f** After 48 h, the mice were tested again with the platform removed, and the speed (left), crossing times (middle), and time in the target quadrant (right) were recorded. **g**, **h** The normalized fEPSP slope (% of baseline) recorded from the CA1–CA3 region in hippocampal slices. *n* = 6. **P < 0.01 compared to controls; ^# #^*P* < 0.01, and ^#^*P* < 0.05 for comparison between the Mn-treated WT mice.

To investigate the effect of Mn on spatial learning and memory in the presence of α-Syn protein, we tested the reference memory of mice in the MWM (Fig. 1a). In the acquisition training phase, between the WT and α-Syn KO control groups, the escape latencies, mean speed, and mean distance exhibited no significant differences (Fig. 1b–e). However, Mn-treated mice showed significantly increased times for escape latency and mean distance compared with control groups, which indicated learning and memory impairments. For the two Mn-treated groups, the data analysis showed that α-Syn KO mice performed better than WT mice with Mn treatment, as revealed by a significant decrease in escape latency and mean distance compared with Mn-treated WT mice on the 6th day (escape latency, *F*_(Mn factor)_ = 153.725, *P* = 0.00, *F*_(genotype)_ = 1.995, *P* > 0.05, *F*_(genotype vs. Mn interaction)_ = 25.772, *P* = 0.00; mean distance, *F*_(Mn factor)_ = 67.656, *P* = 0.00, *F*_(genotype)_ = 2.332, *P* > 0.05, *F*_(genotype vs. Mn interaction)_ = 6.491, *P* = 0.019).

Then, 48 h after the acquisition sessions, the platform was removed. In the probe test, similar crossing times in the platform (target) quadrant were measured for the two control groups, and they spent similar amounts of time in the target quadrant. However, Mn-treated mice showed significantly decreased crossing times and time in the target quadrant during the removal testing session, compared with control groups (Fig. 1f). In addition, Mn-treated α-Syn KO mice were relieved of the effect of Mn. (crossing times, *F*_(Mn factor)_ = 47.619, *P* = 0.00, *F*_(genotype)_ = 3.619, *P* > 0.05, *F*_(genotype vs. Mn interaction)_ = 4.286, *P* = 0.05; time in the target quadrant, *F*_(Mn factor)_ = 333.871, *P* = 0.00, *F*_(genotype)_ = 0.915, *P* > 0.05, *F*_(genotype vs. Mn interaction)_ = 13.265, *P* = 0.002). Our data suggested that α-Syn KO ameliorated the learning and memory impairments induced by Mn.

Deficits in LTP involved in learning and memory may contribute to a number of neurological diseases. In the hippocampus CA3–CA1 region, our results showed that LTP was weakened in Mn-treated mice compared with the control mice, with the maximum inhibition appearing in Mn-treated WT mice. The fEPSP slope change was used to reflect the level of LTP formation. Figure 1g, h indicates that there were no significant differences in the increasing rate of fEPSP slopes between control WT and α-Syn KO mice, as indicated by the changes in the postsynaptic potential slopes. Mn-treated WT mice showed a decreased fEPSP slope growth rate compared with control mice. In addition, α-Syn knockout weakened the effect of Mn (fEPSP slope at 80 min, *F*_(Mn factor)_ = 121.913, *P* = 0.00, *F*_(genotype)_ = 2.811, *P* > 0.05, *F*_(genotype vs. Mn interaction)_ = 209.082, *P* = 0.00). These data reinforced the idea that Mn-induced impairments in spatial memory and synaptic plasticity were related to LTP.

### Mn induces synaptic degeneration in WT and α-Syn KO mice

**Fig. 2:**
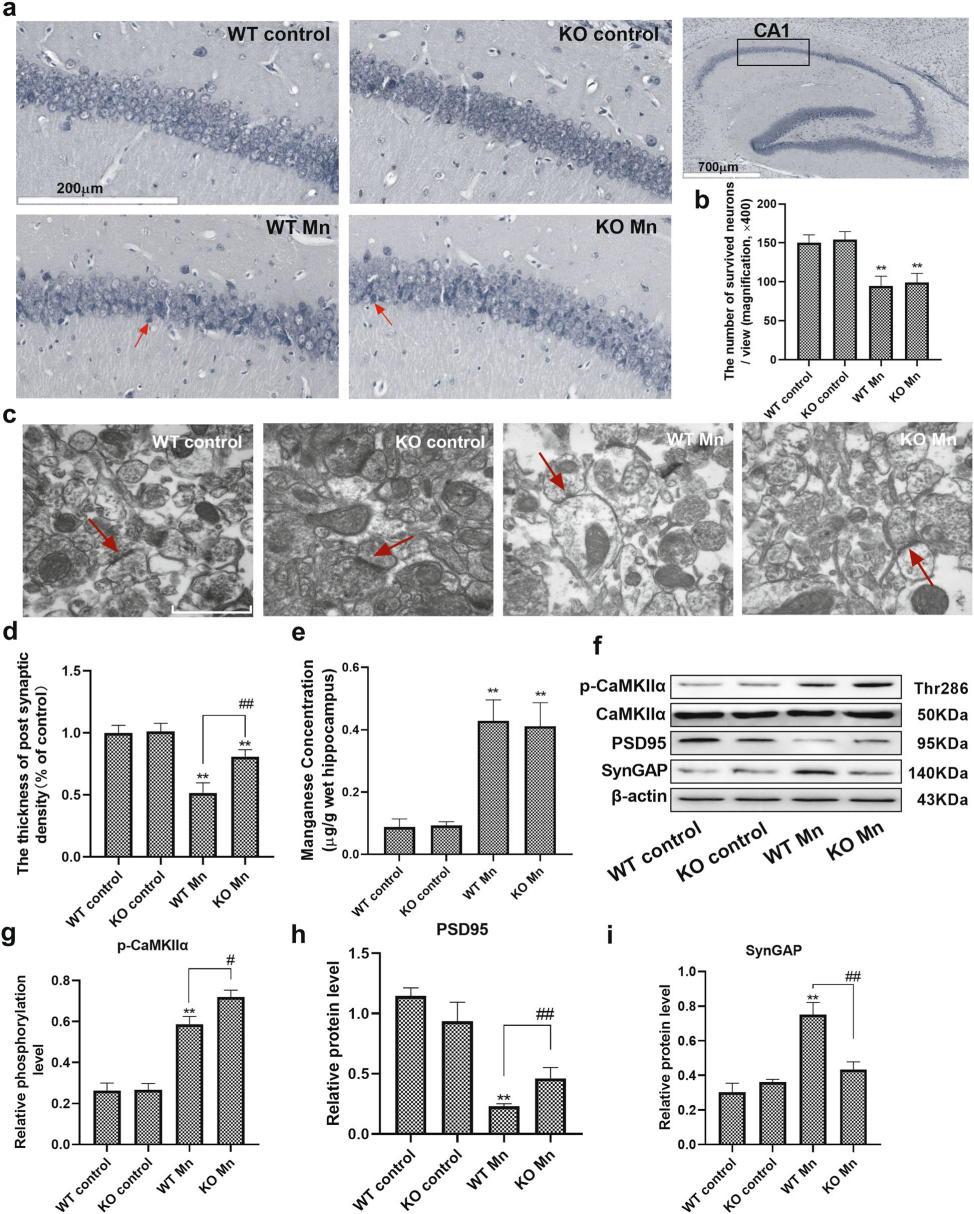
Mn induces synaptic degeneration in WT and α-Syn KO mice. **a**, **b** Representative Nissl staining of the hippocampal CA1 region indicated by the black frame, and the injured neurons are indicated by the red arrows in Mn-treated mice. Bars = 200 μm. **c** Representative transmission electron microscopy image of the synapse ultrastructure. The red arrows indicate the postsynaptic density. Scale bars = 500 nm. **d** Electron microscopy images were quantitated and analyzed by ImageJ. **e** The total levels of manganese were evaluated in the hippocampal region. **f**–**i** The levels of postsynaptic proteins (PSD95, CaMKIIα, and SynGAP) and phosphorylated CaMKIIα were measured by western blotting. *n* = 6. **P < 0.01, and *P < 0.05 compared to controls; ^# #^*P* < 0.01, and ^#^*P* < 0.05 for comparison between the Mn-treated WT mice.

Nissl staining showed that the number of surviving neurons was decreased in the CA1 region in Mn-treated WT and α-Syn KO mice compared with their control mice (WT control 150.1 ± 10.401 vs. WT Mn 94.66 ± 12.662; KO control 154.0 ± 9.585 vs. KO Mn 99.34 ± 11.500, *F*_(Mn factor)_ = 70.668, *P* = 0.00, *F*_(genotype)_ = 0.439, *P* > 0.05, *F*_(genotype vs. Mn interaction)_ = 0.003, *P* > 0.05, Fig. 2a, b). We used transmission electron microscopy (TEM) to observe the pathological features of synaptic ultrastructure. In WT and α-Syn KO mice, normal synaptic ultrastructure was observed in the hippocampus, consisting of a clear synaptic cleft and postsynaptic density (PSD). Electron microscopy data were quantitated and showed that the PSD thickness in the synapses decreased, and the synaptic cleft became blurred in the Mn-treated WT and α-Syn KO mice (WT control 100% ± 0.061 vs. WT Mn 51.6% ± 0.080; KO control 100% ± 0.065 vs. KO Mn 80.8% ± 0.056). However, α-Syn knockout relieved Mn-induced impairment of PSD thickness (*F*_(Mn factor)_ = 1509.49, *P* = 0.00, *F*_(genotype)_ = 1.068, *P* > 0.05, *F*_(genotype vs. Mn interaction)_ = 26.833, *P* = 0.00, Fig. 2c, d).

We measured Mn concentration in the hippocampus to determine how much was absorbed in Mn-treated mice. Statistical comparisons revealed a significant increase in Mn-treated WT and α-Syn KO mice compared with their control counterparts (5.25- and 4.55-fold, respectively, *F*_(Mn factor)_ = 234.795, *P* = 0.00, *F*_(genotype)_ = 0.081, *P* > 0.05, *F*_(genotype vs. Mn interaction)_ = 0.257, *P* > 0.05). In addition, between Mn-treated WT and α-Syn KO mice, statistical analysis showed no difference in Mn concentration (Fig. 2e).

To further investigate synaptic degeneration, we examined the levels of postsynaptic proteins (PSD95, CaMKIIα, and SynGAP) in the hippocampus of WT and α-Syn KO mice. After treatment with Mn, the significant decrease in the level of PSD95 associated with increases in the levels of phosphorylated CaMKIIα and SynGAP were also observed in the hippocampus of WT and α-Syn KO mice, compared with their control counterpart mice. Remarkably, we also found that α-Syn KO mice relieved the Mn-induced degradation of PSD95, phosphorylated CaMKIIα, and reduced the level of SynGAP expression (PSD95 protein, *F*_(Mn factor)_ = 151.670, *P* = 0.00, *F*_(genotype)_ = 0.032, *P* > 0.05, *F*_(genotype vs. Mn interaction)_ = 15.335, *P* = 0.004; p-CaMKIIα protein, *F*_(Mn factor)_ = 364.557, *P* = 0.00, *F*_(genotype)_ = 1.155, *P* > 0.05, *F*_(genotype vs. Mn interaction)_ = 9.767, *P* = 0.014; SynGAP protein, *F*_(Mn factor)_ = 84.375, *P* = 0.00, *F*_(genotype)_ = 1.137, *P* > 0.05, *F*_(genotype vs. Mn interaction)_ = 43.539, *P* = 0.002, Fig. 2f–i). These results showed that α-Syn KO partly relieved Mn-induced synaptic degeneration.

### Mn-dependent enhanced α-Syn expression disturbs the phosphorylation of NMDA receptors

**Fig. 3:**
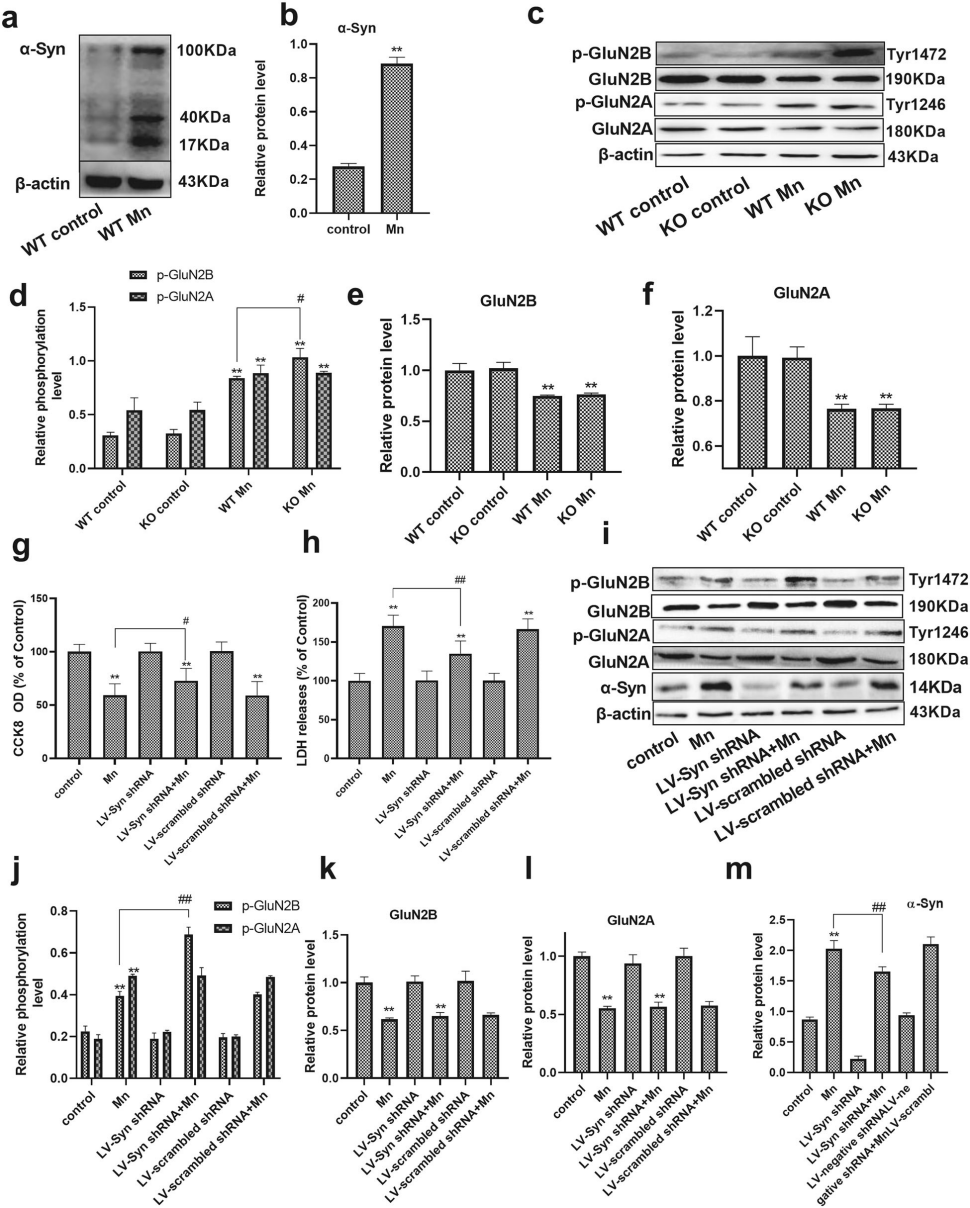
Mn-dependent enhanced α-Syn expression disturbs the phosphorylation of NMDA receptors in vivo and in vitro. **a**, **b** After treatment with Mn, western blotting was used to measure the level of α-Syn overexpression in the hippocampus. **c**–**f** The levels of phosphorylated NMDA receptors (GluN2B and GluN2A) were measured by western blotting in vivo. *n* = 6. **g**, **h** HT22 cells were transfected with LV-α-Syn shRNA, and the cytotoxicity was measured using the CCK-8 assay and lactate dehydrogenase (LDH) release assay. **i**–**m** The levels of phosphorylated NMDA receptors (GluN2B and GluN2A) and α-Syn expression were measured by western blotting in vitro. *n* = 4. **P < 0.01 compared to their control counterparts; ^# #^*P* < 0.01, and ^#^*P* < 0.05 for comparison between the Mn treatment group.

To further confirm that α-Syn overexpression disturbs the phosphorylation of NMDA receptors, α-Syn overexpression and the phosphorylated and total protein levels of GluN2A and GluN2B (the subunits of NMDA receptors) were analyzed in vivo and in vitro. Western blot analysis revealed that the level of α-Syn protein was increased in the hippocampus of Mn-treated WT mice (3.25-fold, *P* < 0.01, Fig. 3a, b). Between the WT and α-Syn KO control groups, statistical analysis showed no differences in the phosphorylated and total protein levels of GluN2A and GluN2B. Following Mn treatment in WT and α-Syn KO mice, we found that the phosphorylation levels of GluN2B were strongly increased (*P* < 0.01, Fig. 3c, d), and that GluN2A and GluN2B total protein levels were strongly decreased compared with their control counterparts (GluN2B protein, *F*_(Mn factor)_ = 70.040, *P* = 0.00, *F*_(genotype)_ = 0.117, *P* > 0.05, *F*_(genotype vs. Mn interaction)_ = 0.355, *P* > 0.05; GluN2A protein, *F*_(Mn factor)_ = 18.771, *P* = 0.003, *F*_(genotype)_ = 0.012, *p* > 0.05, F _(genotype vs. Mn interaction)_ = 0.014, *p* > 0.05, Fig. 3e, f). In addition, Western blot analysis showed that the phosphorylation levels of GluN2B were significantly reduced in Mn-treated WT mice compared with Mn-treated α-Syn KO mice (p-GluN2B protein, *F*_(Mn factor)_ = 487.826, *P* = 0.00, *F*_(genotype)_ = 1.299, *P* > 0.05, *F*_(genotype vs. Mn interaction)_ = 9.962, *P* = 0.013).

To further verify that the downregulation of phosphorylated GluN2B is associated with Mn-dependent α-Syn overexpression, differentiated HT22 (mouse hippocampal neuron precursor) cells were transfected with LV-α-Syn shRNA. The cytotoxicity was measured using the CCK-8 assay, and it was found that there was a significant decrease in Mn-treated cells compared with control cells (*P* < 0.01, Fig. 3g). However, pretreatment with LV-α-Syn shRNA increased cell viability (1.23-fold, *P* < 0.05) and decreased LDH release compared with cells treated with Mn alone (20.78%, *P* < 0.01, Fig. 3h). Similarly, western blot analysis results showed that Mn treatment increased the phosphorylation levels (*P* < 0.01, Fig. 3i, j) and decreased total protein levels of GluN2A and GluN2B compared with control cells (*P* < 0.01, Fig. 3k, l). The phosphorylation levels of GluN2B in cells pretreated with LV-α-Syn shRNA were significantly higher than those in the cells treated with Mn alone (1.74-fold, *P* < 0.01). In addition, the level of α-Syn expression was increased in Mn-treated cells compared with control cells (*P* < 0.01). Pretreatment with LV-α-Syn shRNA decreased the level of α-Syn expression compared with cells treated with Mn alone (*P* < 0.01, Fig. 3m). These results suggested that Mn-dependent enhanced α-Syn expression was responsible for the downregulation of phosphorylated GluN2B.

### BDNF interferes with the interaction of α-Syn and TrkB

**Fig. 4:**
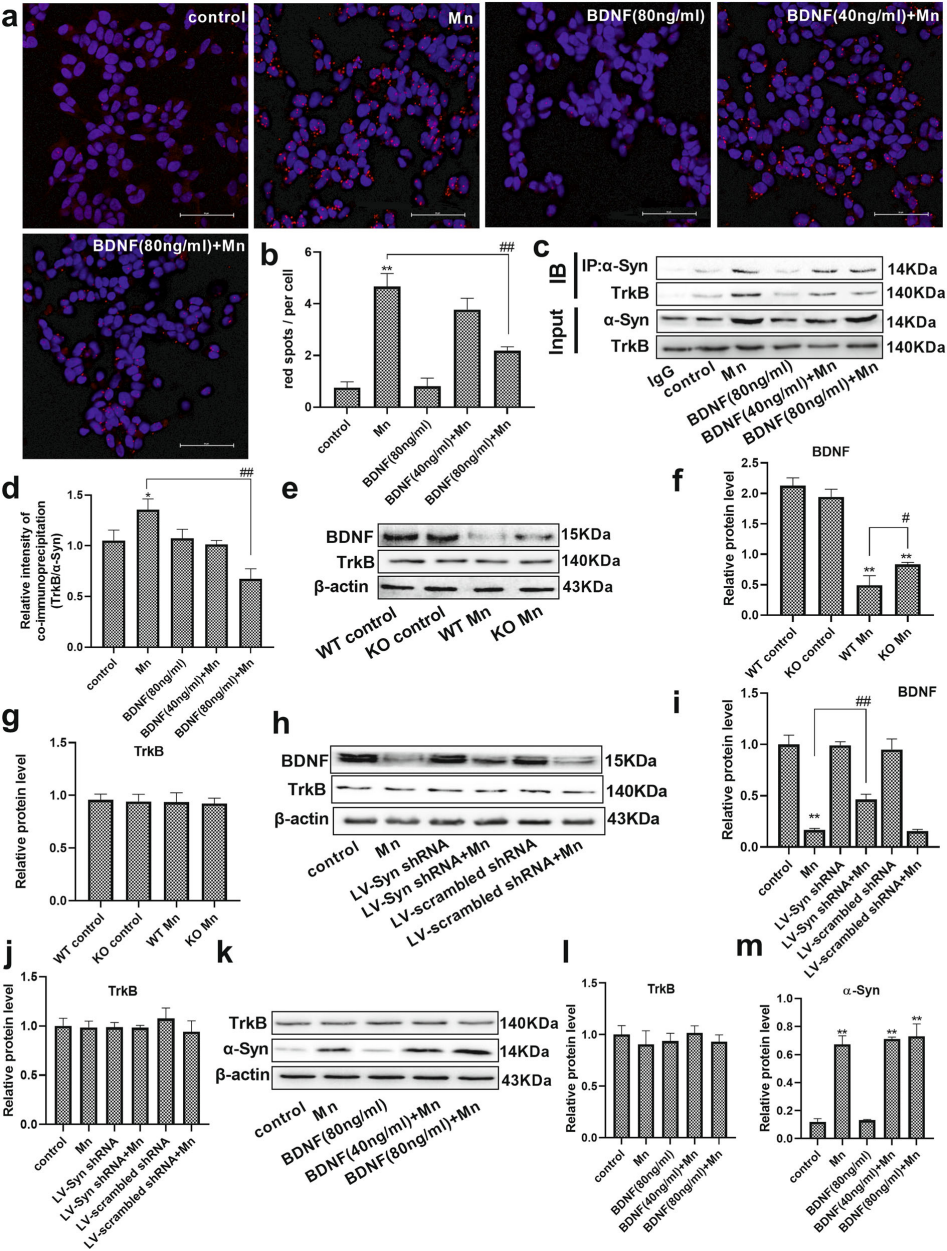
Brain-derived neurotrophic factor (BDNF) interfered with the interaction of α-Syn and TrkB. **a**, **b** After pretreatment with BDNF and Mn in HT22 cells, representative images of PLA show the interaction between α-Syn and TrkB (red spots), and the nucleus was stained in blue (DAPI). Scale bars = 50 μm. **c**, **d** The immunoprecipitation analysis of α-Syn and TrkB is shown. **e**–**g** After treating the mice with Mn, the levels of BDNF and TrkB were evaluated by western blotting. *n* = 6. **h**–**j** For HT22 cells transfected with the LV-α-Syn shRNA, the levels of BDNF and TrkB were evaluated by western blotting after Mn treatment. **k**–**m** After pretreatment with BDNF and Mn in HT22 cells, the levels of TrkB and α-Syn expression were evaluated by western blotting. *n* = 4. **P < 0.01, and *P < 0.05 compared to their control counterparts; ^##^*P* < 0.01, and ^#^*P* < 0.05 for comparison between the Mn treatment group.

We hypothesized that Mn-dependent enhanced α-Syn expression might directly impinge on the BDNF/TrkB pathway. Therefore, we conducted a proximity ligation assay (PLA) using differentiated HT22 cells and found that there was an increasing proximity ligation (PL) signal between α-Syn and TrkB in Mn-treated cells compared with the control cells (6.21-fold, *P* < 0.01, Fig. 4a, b). As expected, we also found that the same tendency was observed in the co-immunoprecipitation (Co-IP) assay (Fig. 4c). The semiquantitative analysis showed that Mn exposure significantly increased the interaction of α-Syn and TrkB compared with the control group (*P* < 0.05, Fig. 4d).

Next, we investigated whether exogenous BDNF could disrupt the interaction of α-Syn and TrkB and protect HT22 cells from damage. The cytotoxicity was measured using the CCK-8 assay and LDH release assay, and it was found that pretreatment with 80 ng ml^−1^ BDNF increased neuronal cell viability (1.37-fold, *P* < 0.01, Supplementary Fig. s1a) and decreased LDH release compared with cells treated with Mn alone (20.05%, *P* < 0.01, Supplementary Fig. s1b). In addition, we found that 80 ng ml^−1^ BDNF pretreatment significantly decreased the interaction of α-Syn and TrkB compared with cells treated with Mn alone (50.55%, *P* < 0.01). These results suggested that α-Syn interacted with TrkB, which could be inhibited by BDNF treatment.

To investigate the effect of α-Syn on BDNF and the TrkB receptor in response to Mn-associated pathology, we next treated mice and differentiated HT22 cells with Mn, LV-α-Syn shRNA, and BDNF, and analyzed the levels of α-Syn, BDNF, and TrkB expression. After treatment with Mn, a significant decrease in the level of BDNF expression was observed in the hippocampus of WT and α-Syn KO mice, compared with their control counterpart mice (BDNF, *F*_(Mn factor)_ = 380.508, *P* = 0.00, *F*_(genotype)_ = 1.277, *P* > 0.05, *F*_(genotype vs. Mn interaction)_ = 3.993, *P* = 0.016). Remarkably, we also found that the effect of Mn on BDNF expression was weaker in α-Syn KO mice compared to WT mice (*P* < 0.05, Fig. 4e, f). However, neither Mn treatment nor α-Syn KO affected the level of TrkB expression (Fig. 4g).

To further verify the effect of Mn-dependent enhanced α-Syn expression on BDNF and the TrkB receptor, cells were transfected with LV-α-Syn shRNA. Similarly, western blot analysis indicated that Mn treatment decreased the level of BDNF expression compared with the control cells (*P* < 0.01, Fig. 4h, i). In addition, after cells were treated with Mn, the level of BDNF in cells pretreated with LV-α-Syn shRNA was significantly higher than that in the normal cells (2.79-fold, *P* < 0.01). Western blot results showed that there was no significant difference in the level of TrkB expression among the experimental groups (Fig. 4j). Furthermore, BDNF pretreatment did not affect the levels of TrkB and α-Syn expression compared with Mn-treated cells (Fig. 4k–m).

### TrkB/Akt/Fyn signaling is required for the phosphorylation of NMDA receptors

**Fig. 5:**
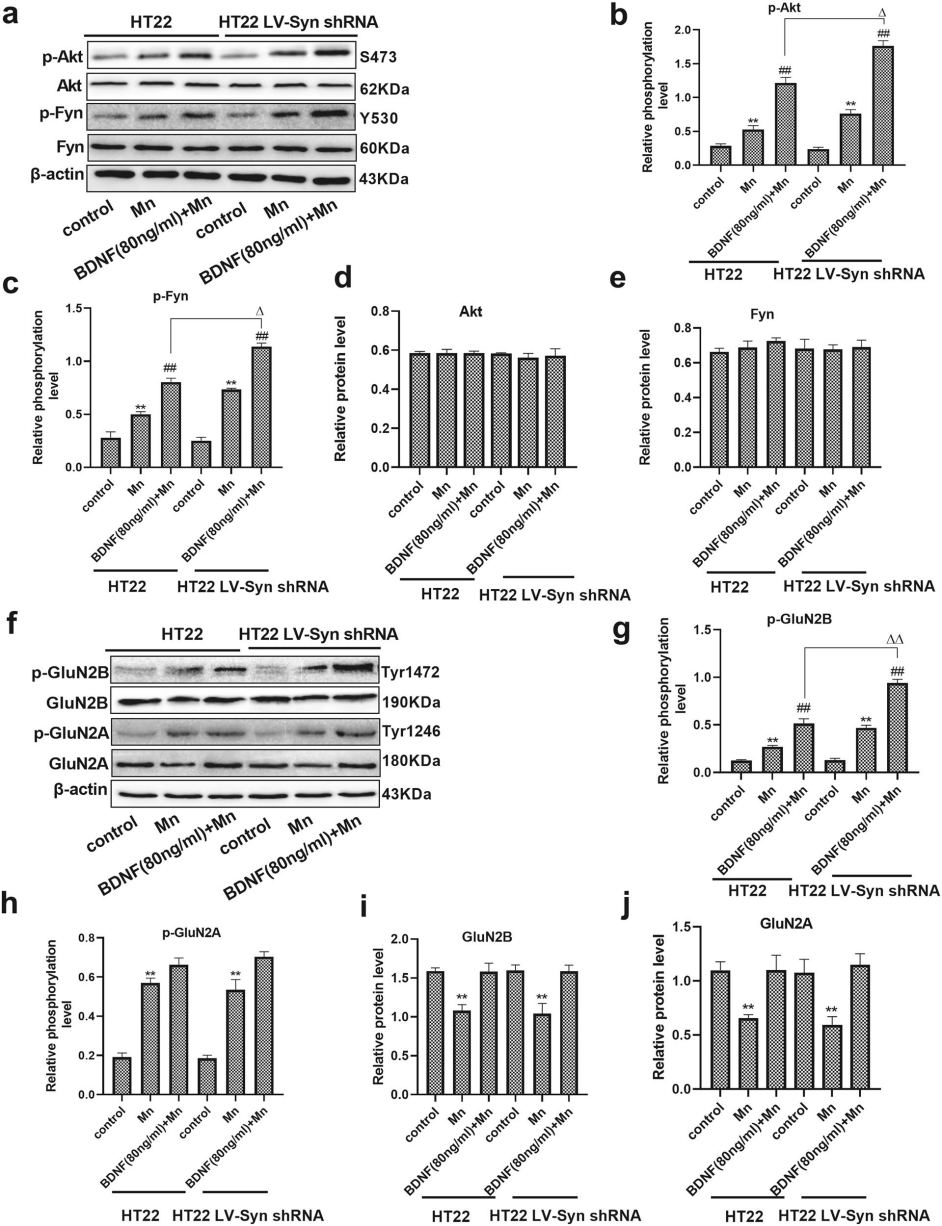
TrkB/Akt/Fyn signaling is required for the phosphorylation of NMDA receptors in vitro. **a**–**e** After normal or transfected HT22 cells were pretreated with brain-derived neurotrophic factor (BDNF) and Mn, the levels of phospho-Akt and phospho-Fyn, and Akt and Fyn expression were evaluated by western blotting. **f**–**j** After normal or transfected HT22 cells were pretreated with BDNF and Mn, the levels of phospho-GluN2B and phospho-GluN2A, and the expression of GluN2B and GluN2A were evaluated by western blotting. *n* = 4. β-actin was used as a loading control. **P < 0.01, and *P < 0.05 compared to their control counterparts; ^##^*P* < 0.01 compared to their Mn-treated cells; ^ΔΔ^*P* < 0.01, and ^Δ^*P* < 0.05 for comparison between their BDNF-pretreated and Mn-treated cells.

To examine the biological effect of α-Syn on BDNF/TrkB signaling, we transfected differentiated HT22 cells with LV-α-Syn shRNA, followed by BDNF and Mn stimulation. In differentiated HT22 cells, exposure to Mn resulted in an increase in the phosphorylation levels of Akt and Fyn compared with their controls (1.87- and 1.79-fold, respectively, *P* < 0.01, Fig. 5a–e). After pretreatment with BDNF, the phosphorylation levels of Akt and Fyn were evaluated. The results clearly showed that phosphorylation levels of these proteins were upregulated compared with those in cells treated with Mn (2.29- and 1.61-fold, respectively, *P* < 0.01). Remarkably, we also found that BDNF-pretreated and Mn-treated cells with LV-α-Syn shRNA showed an increase in the phosphorylation levels of Akt and Fyn compared with BDNF-pretreated and Mn-treated HT22 cells (1.45- and 1.41-fold, respectively, *P* < 0.05), and also a decrease compared with alone BDNF-treated cells with LV-α-Syn shRNA (*P* < 0.01, Supplementary Fig. s2a–e).

To further verify that BDNF/TrkB signaling contributed to the phosphorylation of GluN2A and GluN2B, BDNF was added to Mn-treated HT22 cells. Western blot analysis results showed that in BDNF-pretreated and Mn-treated cells with LV-α-Syn shRNA group, the phosphorylated protein levels of GluN2B were upregulated (1.82-fold, *P* < 0.01, Fig. 5f–h) compared with BDNF-pretreated and Mn-treated HT22 cells, and the total protein levels of GluN2A and GluN2B were significantly decreased (*P* < 0.01, Fig. 5i, j) compared with their control counterparts. However, statistical analysis showed no difference in the phosphorylation level of GluN2A in BDNF-pretreated normal or transfected HT22 cells. Taken together, these results indicate that Mn-dependent enhanced α-Syn expression was responsible for the downregulation of phosphorylated GluN2B through the inhibition of BDNF-mediated TrkB signaling.

## Discussion

The hippocampus, as a long-term memory and learning region, is one of the most sensitive areas to Mn overexposure in the brain^20^. In this regard, this study investigated the mechanisms of learning and memory impairments via modulating the signaling pathway factors in the hippocampus after exposure to Mn. In this study, we highlight two significant observations. One is that Mn causes impairments in learning and memory and the inhibition of LTP, which are related to the phosphorylation levels of NMDA receptors. The other is that Mn-dependent enhanced α-Syn expression and BDNF reduction disturbed TrkB signaling, resulting in the downregulation of phosphorylated GluN2B.

In this study, we found that no difference existed between genotypes in spatial memory function in the control groups. However, when treated by Mn, both WT and α-Syn KO mice showed worse performance in both the acquisition and removal phase compared with the control groups. These results may represent a manifestation of learning and memory impairments in Mn-treated mice. In addition, our results indicated that α-Syn KO might ameliorate spatial memory impairment caused by Mn. However, there was no difference in speed between genotypes in the training or testing phase, which partially indicated the emotional influences on the outcomes of the water maze test. The formation of LTP is considered as a potential cellular mechanism in learning and memory processes. According to the findings of previous studies^21^, our study revealed that α-Syn KO did not influence the LTP under normal conditions. This might be because LTP is mainly caused by postsynaptic mechanisms in the CA3–CA1 pathway, but the α-Syn effects on synaptic transmission are predominantly presynaptic^22^. However, Mn exposure promotes α-Syn secretion in exosomal vesicles, which subsequently evokes proinflammatory and neurodegenerative responses in both cell culture and animal models^23^, and thus, it can be supposed that the exosome-mediated, cell-to-cell transmission of α-Syn during exposure to Mn may inhibit the formation of LTP leading to the regression of spatial memory in Mn-treated mice.

The greater selective neuron loss and synaptic degeneration in the hippocampus after Mn treatment may account for the spatial memory and LTP impairments. As shown in this study, surviving neurons were stained by Nissl in the CA1 region, which was important for LTP induction, and these were significantly decreased in Mn-treated WT and α-Syn KO mice. In addition, the synaptic ultrastructural alterations produced by the neurotoxic effect of Mn in the hippocampus were the decrease in the thickness of PSD, while α-Syn knockout weakened the effect of Mn. An elevation of Mn concentration in the hippocampus was detected in Mn-treated mice. Consistent with this notion, the elevation of Mn concentration in the hippocampus resulted in deficits in spatial memory, loss of neurons, and impairment in hippocampal LTP^21,24^. However, between Mn-treated WT and α-Syn KO mice, no significant difference was shown in neuronal injury or Mn concentration in the hippocampus. The major reason for this is that multiple factors are involved in Mn-induced neurotoxicity, including oxidative injury, mitochondrial dysfunction, and neuroinflammation^1^.

A few studies have proved that postsynaptic signaling proteins, such as PSD95 Ca^2+^/calmodulin-dependent protein kinase II with alpha chain (CaMKIIα), and synaptic Ras GTPase-activating protein (SynGAP) associated with NMDA receptors in the hippocampus, play a vital role in LTP^11^. Downregulation of the levels of PSD95 and phosphorylated CaMKIIα related to enhancing the expression of SynGAP upon exposure to Mn as shown in this study could be associated with an alteration in LTP. Consistent with the findings in LTP impairments, α-Syn KO relieved Mn-induced the degradation of PSD95, phosphorylated CaMKIIα, and reduced the level of SynGAP expression.

The N-methyl-D-aspartate (NMDA) receptor is a heteromeric complex of three subunits differentially distributed in the brain. Some studies showed that phosphorylation of the NMDA receptor 2 subunit (NR2), especially GluN2A and GluN2B, regulates the activity of NMDA receptors and induces LTP formation, memory formation, and synaptic plasticity^25^. Our previous study also showed that calpain was activated by Mn-mediated Ca^2+^ influx and exhibited selectivity in the degradation of GluN2A and GluN2B^17,26^. In this study, a decrease in the total protein levels of both GluN2A and GluN2B was observed on Mn exposure both in vivo and in vitro, and these results were consistent with our previous findings^27^. In particular, phosphorylated GluN2B has been shown to play a vital role in NMDA receptor activation. In this study, Mn-treated mice exhibited elevation of phosphorylated NMDA receptor, while the phosphorylation level of GluN2B was significantly lower in Mn-treated WT mice compared with Mn-treated α-Syn KO mice; this downregulation caused the decline of LTP in Mn-treated WT mice. Additionally, HT22 cells transfected with LV-α-Syn shRNA exhibited an elevation of phosphorylated GluN2B induced by Mn treatment. The phosphorylation level of GluN2A did not differ between normal and LV-α-Syn shRNA-transfected cells. These results suggested that Mn-dependent enhanced α-Syn expression participated in NMDA receptor activation, especially through the phosphorylation of GluN2B.

BDNF is a potent dopaminergic neurotrophin involved in regulating NMDA receptor-dependent synaptic plasticity^28^. In this study, we found that Mn treatment decreased the level of BDNF expression in both WT and α-Syn KO mice. In addition, there was a more significant decline in BDNF of Mn-treated WT mice, while Mn treatment resulted in α-Syn protein overexpression. These results are consistent with previous findings^29^ and suggest that α-Syn overexpression might account for the downregulation of BDNF. Therefore, it is highly possible that BDNF downregulation might have a profound effect on the inhibition of BDNF/TrkB signaling. Studies have indicated that many important cellular processes, such as synaptic vesicle recycling, intracellular trafficking, mitochondrial energetics, lysosomal activity, and autophagy, are all susceptible to α-Syn toxicity^30,31^, suggesting a multifaceted mode of action of neuronal toxicities by the accumulation of α-Syn. Due to the well-documented correlation between aberrant α-Syn accumulation and neuronal degeneration, we hypothesized that pathological α-Syn might directly impinge on the BDNF/TrkB pathway. To test this hypothesis, we conducted PLA and Co-IP assay and found that α-Syn selectively interacted with TrkB, which was relieved by exogenous BDNF pretreatment. Hence, this innovative finding provides insight into a pathological role(s) of α-Syn in mediating BDNF/TrkB signal transduction and may represent an obscure mechanism by which a-Syn contributes to spatial memory and LTP impairments.

BDNF and the activation of its high-affinity receptor TrkB play a vital role in the neuroprotection mediated by the PI3K/Akt signaling pathway. The PI3K/Akt signaling pathway is commonly activated by oxidative stress against nerve cell death^32^. In our study, overexposure to Mn triggered the PI3K/Akt pathway through upregulating the phosphorylation level of Akt, which then inhibited the transcription function of apoptosis genes and negatively regulated apoptosis, leading to cell survival^33^. Interestingly, a significant increase in the level of p-Akt expression was observed in cells transfected with LV-α-Syn shRNA, followed by BDNF stimulation. These findings indicated that α-Syn disturbed BDNF/TrkB signal transduction. Fyn, a member of the Src family, has been proved to mediate phosphorylation and upregulate GluN2B-containing NMDA receptors, leading to enhanced receptor channel activity^34^. As a downstream signaling molecule of Akt, Fyn kinase is essential for synaptic plasticity^35,36^. In our study, a significant increase in the level of p-Fyn had been observed in cells transfected with LV-α-Syn shRNA, followed by BDNF stimulation compared with Mn-treated HT22 cells, which was consistent with the phosphorylation of GluN2B. These results are identical to previous findings^37^, and suggest that Fyn-mediated phosphorylation of GluN2B acts on the tyrosine 1472 (Tyr1472) site and plays an important role in hippocampal LTP and hippocampus-dependent memory formation. However, the deletion of α-Syn does not entirely annul the effects of Mn on Fyn-mediated phosphorylation of GluN2B. This may be explained by other mechanisms of Mn including accelerating BDNF protein degradation and reducing binding to TrkB^3,38^, which contribute to Mn-induced neurotoxicity.

## Conclusion

**Fig. 6:**
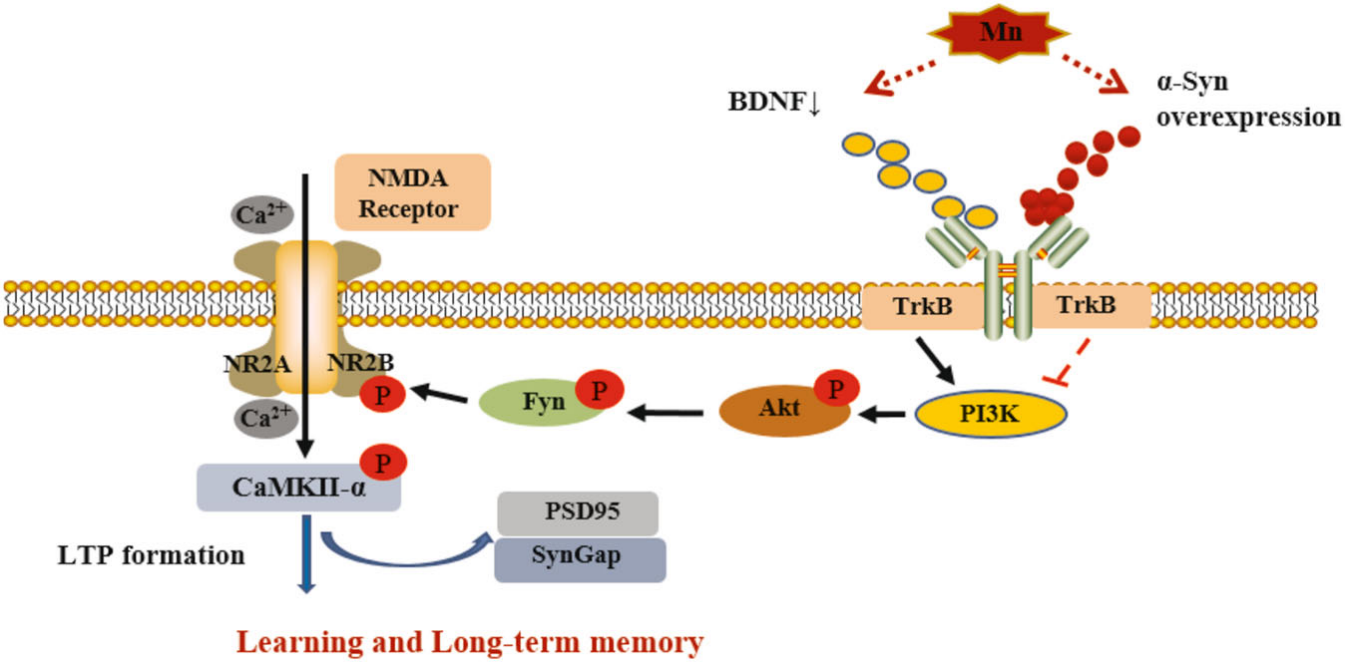
The proposed mechanism of Mn-induced spatial memory and long-term potentiation (LTP) impairments. Mn-induced α-Syn protein overexpression contributes to further exacerbate brain-derived neurotrophic factor (BDNF) protein-level reduction and to inhibit TrkB/Akt/Fyn signaling, thereby disturbing Fyn-mediated phosphorylation of the NMDA receptor GluN2B subunit at tyrosine.

This study provided evidence that Mn-induced α-Syn protein overexpression contributes to further exacerbate BDNF protein-level reduction and to inhibit TrkB/Akt/Fyn signaling, thereby disturbing Fyn-mediated phosphorylation of the NMDA receptor GluN2B subunit at tyrosine (Fig. 6). In KO α-Syn mice treated with Mn, spatial memory and LTP impairments were less pronounced than in WT mice. However, the same robust neuronal death was observed in the face of Mn-induced neurotoxicity.

## Supplementary information

Supplementary Figure 1

Supplementary Figure 2

Supplementary Figure Legends

## Data Availability

All data generated or analyzed during this study are included in this published article.
