# Supplementary figures and images for: Alpha-synuclein is involved in manganese-induced spatial memory and synaptic plasticity impairments via TrkB/Akt/Fyn-mediated phosphorylation of NMDA receptors
Source: Cell Death Dis. 2020 Oct 8;11(10):834. doi: 10.1038/s41419-020-03051-2 (PMC7545185; doi:10.1038/s41419-020-03051-2)

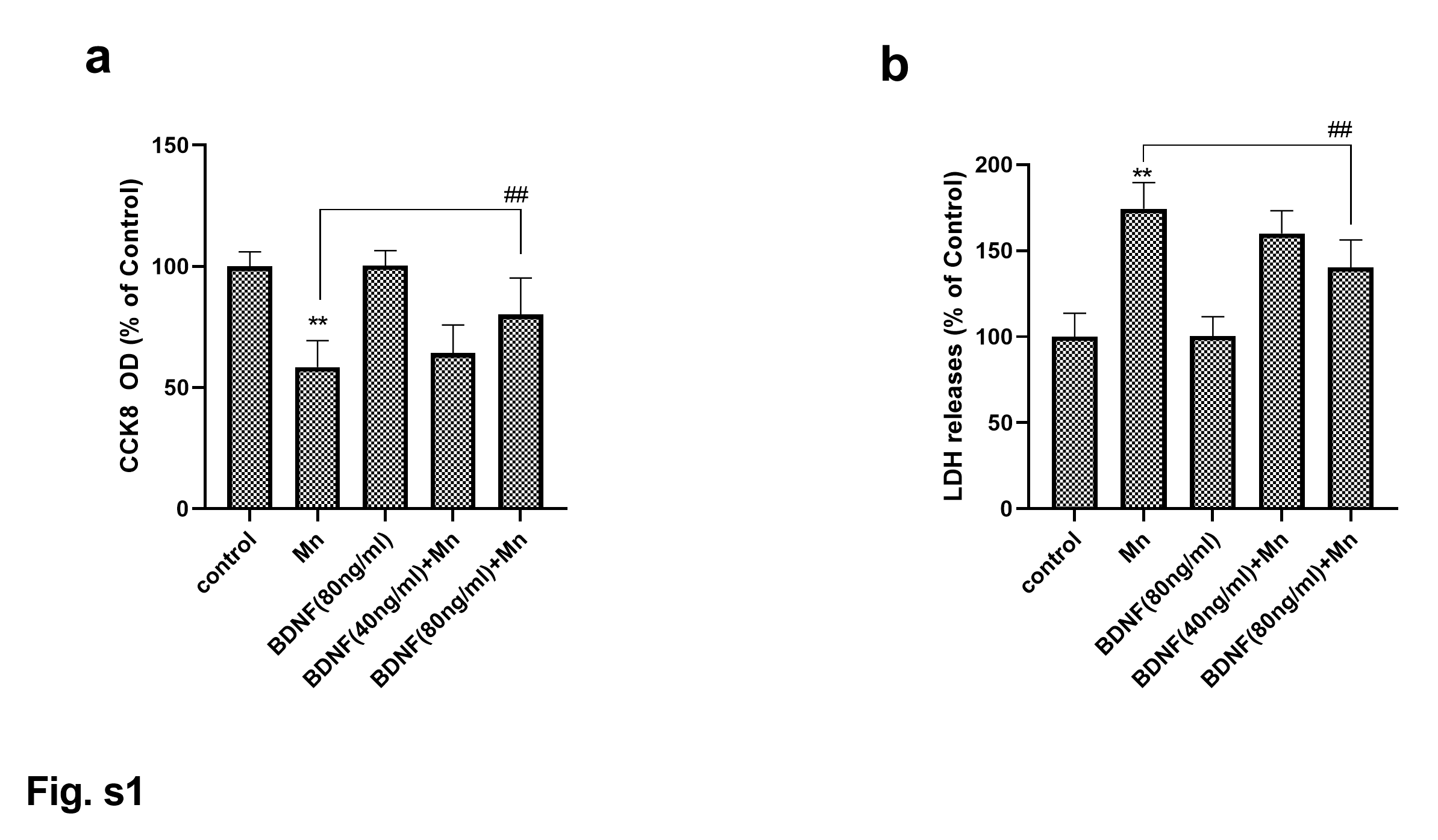

Supplement: Supplementary file 1 — Supplementary Figure 1 [file 41419_2020_3051_MOESM1_ESM.tif]

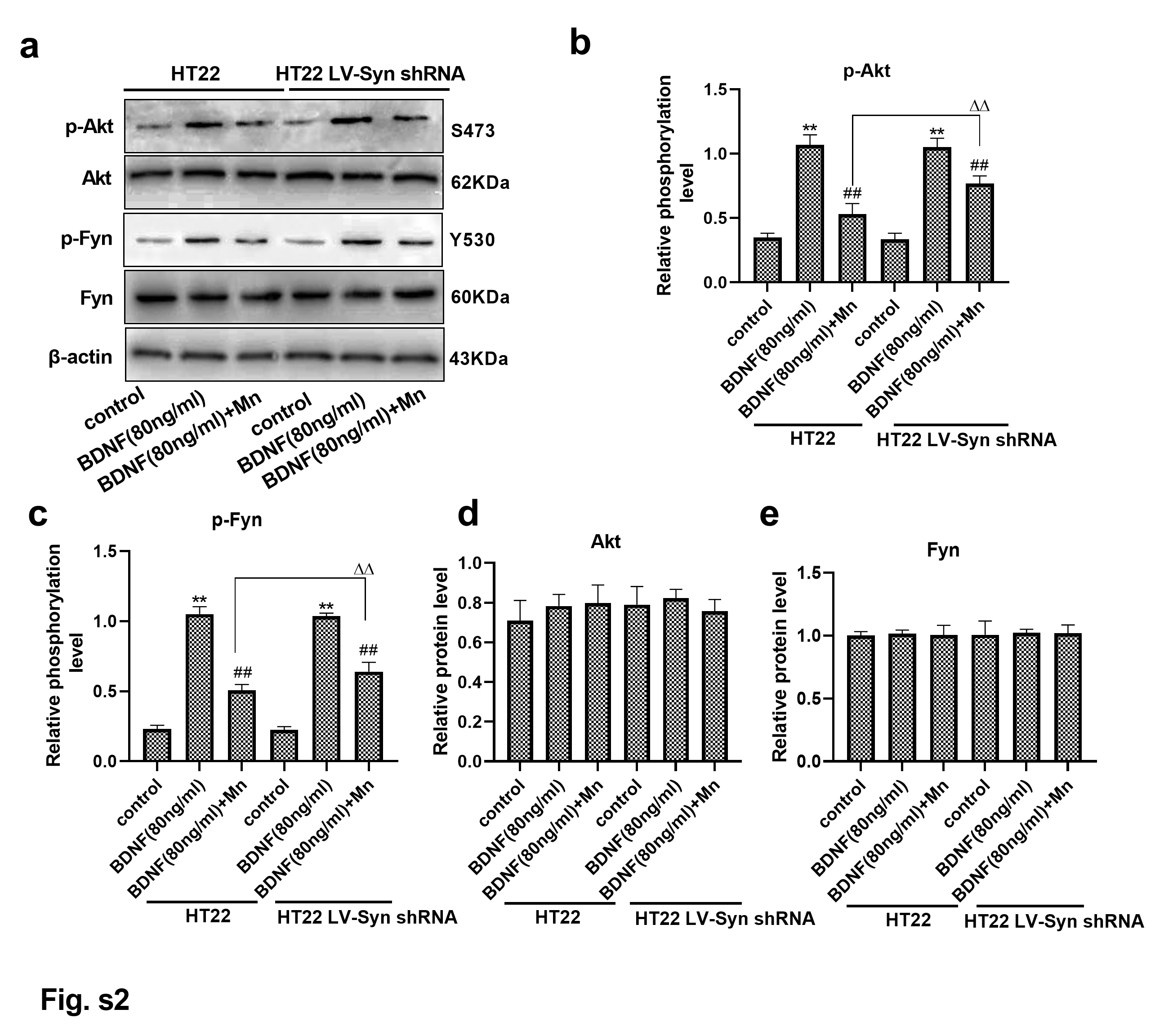

Supplement: Supplementary file 2 — Supplementary Figure 2 [file 41419_2020_3051_MOESM2_ESM.tif]
