# Supplementary material for: Alpha-synuclein is involved in manganese-induced spatial memory and synaptic plasticity impairments via TrkB/Akt/Fyn-mediated phosphorylation of NMDA receptors
Source: Cell Death Dis. 2020 Oct 8;11(10):834. doi: 10.1038/s41419-020-03051-2 (PMC7545185; doi:10.1038/s41419-020-03051-2)
Supplement: Supplementary file 3 — Supplementary Figure Legends [file 41419_2020_3051_MOESM3_ESM.docx]

**Alpha-synuclein is involved in manganese-induced** **spatial memory and synaptic plasticity impairments via TrkB/Akt/Fyn-mediated phosphorylation of NMDA receptors**

Running Title: Mn-induced α-Syn disturbs TrkB-mediated p-GluN2B

Zhuo Ma, Kuan Liu, Xin-Ru Li, Can Wang, Chang Liu, Dong-Ying Yan, Yu Deng, Wei Liu, and Bin Xu*

Department of Environmental Health, School of Public Health, China Medical University, No.77 Puhe Road, Shenyang North New Area, Shenyang, Liaoning Province, 110122, People’s Republic of China

* Correspondence to: Bin Xu E-mail: [bxu10@cmu.edu.cn](mailto:bxu10@cmu.edu.cn). The telephone number is +86-18940062096.

**Supplementary Figure Legends**

**Fig. s1** The cytotoxicity was measured after pretreatment with BDNF in HT22 cells.

(a,b) CCK-8 assay and LDH release were used to assay neuronal cell viability. n = 4. ***p* < 0.01 compared to their control counterparts; # # *p* < 0.01 compared to their Mn-treated cells.

**Fig. s2** The levels of relative phosphorylation and total protein expression in TrkB/Akt/Fyn signaling *in vitro*.

(a-e) After normal or transfected HT22 cells were pretreated with BDNF and Mn, the levels of phosopho-Akt, phosopho-Fyn, Akt and Fyn expression were evaluated by western blotting. n = 4. β-actin was used as a loading control. ***p* < 0.01 compared to their control counterparts; ^# #^ *p* < 0.01 compared to their BDNF-pretreated cells; ^ΔΔ^ *p* < 0.01 for comparison between their BDNF-pretreated and Mn-treated cells.
